# Pathophysiological Roles of PPAR**γ** in Gastrointestinal Epithelial Cells

**DOI:** 10.1155/2008/148687

**Published:** 2008-07-06

**Authors:** Brian M. Necela, E. Aubrey Thompson

**Affiliations:** Department of Cancer Biology, Mayo Clinic Comprehensive Cancer Center, Jacksonville, FL 32224, USA

## Abstract

Although the highest levels of PPAR*γ* expression in the body have been reported in the gastrointestinal epithelium, little is known about the physiological functions of that receptor in the gut. Moreover, there is considerable controversy concerning the effects of thiazolidinedione PPAR*γ* agonists on the two major diseases of the gastrointestinal track: colorectal cancer and inflammatory bowel disease. We will undertake to review both historical and recently published data with a view toward summarizing what is presently known about the roles of PPAR*γ* in both physiological and pathological processes in the gastrointestinal epithelium.

## 1. INTRODUCTION

Peroxisome proliferator-activated receptor-gamma (PPAR*γ*, NR1C3) has been described as a master regulator of adipocytes differentiation [1]; and since the discovery that the insulin-sensitizing thiazolidinedione drugs are PPAR*γ* agonists [2], the role of this receptor in adipogenesis has been studied in detail. PPAR*γ* is also abundant in the gastrointestinal tract, where it is highly expressed in epithelial cells [3, 4]. High-level expression of PPAR*γ* in the gut epithelium suggests some important physiological role in the gut, although this role is not well understood. From the standpoint of pathology, PPAR*γ* has been implicated in both transformation and inflammation in the gut. However, there are conflicting data concerning the efficacy and even the safety of PPAR*γ* agonists in clinical management of gastrointestinal cancer and inflammation. We will undertake in this review to summarize both historical and recent data that relate to three questions: What is the physiological role of PPAR*γ* in gastrointestinal epithelial cells? Is PPAR*γ* a colon cancer suppressor? And what is the role of PPAR*γ* in inflammation?

## 2. THE PHYSIOLOGICAL ROLE OF PPAR*γ* IN GASTROINTESTINAL EPITHELIAL CELLS

PPAR*γ* is expressed in epithelial cells of both the large and small intestines [3, 4]. Relatively lower levels of expression are observed in the small intestine, which appears to express PPAR*γ* at more or less uniform abundance from the duodenum to the caecum [5]. Very high levels of PPAR*γ* are expressed in the proximal colon, with somewhat lower levels observed in the mid and distal colon [5, 6]. The highest levels of PPAR*γ* in the body are observed in highly differentiated lumenal epithelial cells of the proximal colon. It has also been observed that PPAR*γ* is induced when Caco2 cells undergo differentiation in culture [6], leading to the suggestion that induction of PPAR*γ* is associated with differentiation of colonic epithelial cells. However, a careful analysis of PPAR*γ* expression in the colon suggests that this conclusion is correct only within a limited context [5]. Basal epithelial cells of proximal crypts are highly differentiated, yet express significantly lower levels of PPAR*γ* than lumenal epithelial cells from the same crypts. Furthermore, PPAR*γ* expression in the distal colon is more or less uniform throughout the crypts, with slightly higher levels of expression observed in the less differentiated basal crypt cells, rather than the more highly differentiated lumenal epithelial cells [5, 7]. Thus, it is not generally the case that induction of PPAR*γ* is associated with differentiation of colonic epithelial cells. It is, however, the case that all colonic epithelial cells express significant levels of PPAR*γ*. This is an important point, since some of the effects of PPAR*γ* are manifest in the transit amplifying cells that support renewal of the colonic epithelium. Whether PPAR*γ* is expressed in colonic stem cells remains an open question.

A significantly different pattern of PPAR*γ* expression is observed in the epithelium of the small intestine. Transit amplifying cells within the crypts of Lieberkühn express little or no PPAR*γ* [8, 9]. Instead, this receptor is induced at the crypt/villus junction, where small intestinal epithelial cells undergo differentiation into mature villus epithelial cells. Thus, in the small intestine it is unambiguously the case that induction of PPAR*γ* is associated with differentiated function, and it has been reported that PPAR*γ* collaborates with Hic5 to promote differentiation of embryonic small intestinal epithelial cells [10].

Efforts to understand the role of PPAR*γ* in differentiated function of gut epithelial cells have been hindered by the lack of good cellular models to study the mechanisms of action of PPAR*γ* in culture. There are no nontransformed colonic epithelial cell lines, and primary colonic epithelial cells have proved to be difficult, if not impossible, to maintain in culture for even a few hours. There are several nontransformed epithelial cell lines derived from the rat embryonic small bowel (e.g., RIE1 and IEC6), but these cells are derived from proliferative crypt cells and, like the transit amplifying cells from which they were derived, these cells express no PPAR*γ*. It has been possible to engineer RIE1 cells to express PPAR*γ*, thereby modeling the transition that occurs at the crypt/villus junction [8, 11]. Activation of PPAR*γ* in such cells results in irreversible withdrawal form the cell cycle, promotes motility, and reduces cellular adhesion.

Genomic profiling indicates that PPAR*γ* targets in such cells fall into four major functional cohorts [8]. The largest of these cohorts consists of genes that are involved in metabolism, and a large proportion of lipid transport and metabolism genes are evidenced within this group. This observation is consistent with our understanding of the metabolic role of PPAR*γ* in other tissues [12–16]. A second cohort of PPAR*γ* target genes was ontologically linked to signal transduction. The data suggest that there is extensive crosstalk between PPAR*γ* and other signaling pathways within intestinal epithelial cells. A third cohort of genes was linked to proliferation, consistent with the observation that activation of PPAR*γ* within these cells results in inhibition of culture growth and irreversible withdrawal from the cell cycle. Somewhat surprisingly, the forth functional cohort of PPAR*γ* target genes was ontologically linked to cellular motility and adhesion. Such processes have not been generally thought of as linked to activation of nuclear receptors. Nevertheless, activation of PPAR*γ* in intestinal epithelial cells potently induces cellular motility, through a mechanism that involves Rho family GTPases and MAPK activation [11]. Renewal of the intestinal epithelium is tightly coupled to migration of differentiated epithelial cells from the crypts to the villus tips, and the observation that PPAR*γ* regulates intestinal epithelial cell motility provides a very important clue into the potential physiological role of this receptor in the gastrointestinal epithelium.

Genomic analysis of PPAR*γ* targets in colonic epithelial cells isolated from thiazolidinedione-treated mice indicates that, in general, similar processes are regulated in epithelial cells from the colon and small intestine [5]. Major ontological cohorts were identified that link PPAR*γ* activation to metabolism, signal transduction, and migration/motility. In contrast to the results obtained with intestinal epithelial cells in culture, no proliferative cohort of PPAR*γ* target genes was identified in colonic epithelial cells isolated from thiazolidinedione-treated mice. This result was unanticipated since such drugs significantly inhibit BrdU incorporation into both proximal and distal colonic epithelial cells. Failure to detect a cohort of proliferation-related PPAR*γ* target genes in vivo is probably attributable to the fact that only a small subpopulation of cells is involved in proliferation in the colonic epithelium, such that the contribution of RNA from such cells is diluted by the much larger postmitotic population. Genomic studies of this sort are, therefore, useful for analysis of PPAR*γ* effect on the differentiated, postmitotic epithelial cells; but such studies are unlikely to reveal much information about the effects of PPAR*γ* on proliferative colonic epithelial cells, which are presumably the targets for transformation.

PPAR*γ* expression and distribution are very different in the proximal and distal colonic epithelium, implying that this receptor may have different functions in the proximal and distal colon. Genomic analysis indicates that the majority of PPAR*γ* target genes are expressed in both proximal and distal epithelial cells [5], suggesting that there is substantial overlap between the physiological functions of PPAR*γ* in these tissues. However, a subset of PPAR*γ* target genes is restricted to the proximal colon, and a second subset is expressed predominantly in the distal colon. Intriguingly, the proximal PPAR*γ* target genes are all induced by thiazolidinediones, whereas the distal target genes are all repressed. The significance of this observation is unknown at this time. However, the observation that PPAR*γ* represses genes that are differentially expressed in the distal colon is consistent with the hypothesis that PPAR*γ* may suppress differentiated function in that tissue. This hypothesis tends to contradict a large number of observations to the effect that PPAR*γ* promotes differentiated function, and additional studies are needed to confirm this unanticipated observation.

In summary, the lack of appropriate cellular models to study the mechanism of action of PPAR*γ* in nontransformed gut epithelial cells has significantly impaired our ability to understand the role of this receptor in gut physiology. Nevertheless, genomic analyses of genetically engineered intestinal epithelial cells and colonic epithelial cells isolated from thiazolidinedione-treated mice have provided important clues. The data suggest that PPAR*γ* is a potent metabolic regulator in gut epithelial cells. This suggestion is obviously consistent with what we know about PPAR*γ* in mesenchymal cells. There is a strong suggestion that PPAR*γ* is involved in extensive crosstalk with other signal transduction pathways, suggesting that this receptor plays an important role in integrating the physiological response to a wide variety of extracellular signals in vivo. Finally, both genomic and cellular data indicate that PPAR*γ* plays a very important role in regulating cellular motility, which is one of the major differentiated functions of intestinal epithelial cells. The challenge at this time is to put the information we have into a physiological context and to use these data to understand the role of PPAR*γ* in malignant transformation of gastrointestinal epithelial cells.

## 3. PPAR*γ* AS A COLON CANCER SUPPRESSOR

The evidence in support of PPAR*γ* as a colon cancer suppressor is based upon a relatively small number of observations, and not all of these observations are consistent with each other. The most compelling data come from studies using the azoxymethane (AOM)-treated rodent model, which has been widely studied as a model of sporadic colon carcinogenesis. Two initial studies in AOM-treated rats used troglitazone, a relatively weak PPAR*γ* agonist [17, 18]. The endpoint in these experiments was aberrant crypt foci (ACF), rather than colon tumors. Nevertheless, both of these studies indicated that troglitazone potently inhibits ACF formation, thereby presumably reducing the risk of subsequent tumor formation in rats. It was subsequently shown that troglitazone, pioglitazone, and rosiglitazone inhibit ACF formation in AOM-treated Balb/c mice [19]. This was the first study that demonstrated that thiazolidinediones inhibit colon tumor formation, in addition to ACF formation, in the AOM model of colon carcinogenesis. This observation was consistent with the report that whole animal hemizygous knockout of PPAR*γ* suppressed AOM-mediated colon tumor formation in mice [7]. It has subsequently been reported that RS5444, a very high-affinity third generation thiazolidinedione, inhibits ACF formation and blocks tumor formation in AOM-treated C57BL/6 mice [5, 20]. Overall, these data unambiguously indicate that PPAR*γ* inhibits some very early step in transformation of colonic epithelial cells in AOM-treated rodents.

In contrast to the data cited above, two reports have concluded that thiazolidinediones induce caecal tumors in mice [21, 22]. These reports have not been independently confirmed, and other investigators have not observed such an effect. However, this may reflect the fact that caecal tumors were observed only in mice that had received very high concentrations of thiazolidinediones for very long periods of time [21, 22]. The pathological significance of these observations is unclear at this time. Caecal tumors are very rare in both mice and humans, and the concentrations of thiazolidinediones that were used in these experiments were very likely far beyond any dose that would be tolerated in humans, in which peripheral edema is the dose limiting response to such drugs. Nevertheless, the potential significance of these disturbing observations warrants additional consideration.

The major controversy in the PPAR*γ* field has revolved around two high-profile papers that reported increased colon tumor formation in APC^+/Min^ mice treated with thiazolidinediones [23, 24]. One of these papers reported a significant increase in colon tumor size in APC^+/Min^ mice treated with either troglitazone or rosiglitazone. The companion paper reported a slight, but significant, increase in the number of colon tumors in APC^+/Min^ mice treated with troglitazone. No increase in tumor size was observed in the second report, which was, therefore, not entirely consistent with the first. Moreover, two subsequent reports failed to reproduce the effect of thiazolidinediones in APC^+/Δ1309^ or APC^+/Min^ mice [25, 26]. It was also reported that whole animal hemizygous knockout of PPAR*γ* had no affect on tumor number or size in APC^+/Min^ mice [7], an observation that is at variance with the notion that PPAR*γ* promotes tumor formation in mice that contain activating germ line mutations in the Wnt/APC/*β*-catenin pathway. It has recently been reported that biallelic knockout of PPAR*γ* in colonic epithelial cells promotes tumor formation in APC^+/Min^ mice [27], indicating that PPAR*γ* is, in fact, acting to suppress tumor formation in the Min mouse. On the whole, the evidence no longer supports the hypothesis that activation of PPAR*γ* promotes tumor formation in mice with germ line APC mutations.

A recent report describes the effects of PPAR*γ* agonists in pre-established tumors in AOM-treated mice [20]. Such tumors invariably contain somatic mutations that activate the Wnt/APC/*β*-catenin signaling pathway [28]. Thiazolinedinediones had no effect on growth or incidence of colon tumors when the drug was given after tumor formation had occurred [20]. However, activation of PPAR*γ* under these circumstances had a profound inhibitory effect on tumor progression. This effect was most strikingly apparent in the development of carcinoma in situ, which was detected in about 1/3 of the control tumors but was never observed in thiazolidinedione-treated tumors. Since formation of carcinoma in situ involves invasion of the surrounding stoma, it is tempting to speculate that this observation indicates that PPAR*γ* inhibits invasion in vivo, consistent with several reports that indicate that invasion by human colon cancer cell lines in culture is inhibited by PPAR*γ* [9, 29]. Notably, activation of PPAR*γ* in pre-established tumors had no significant effect on BrdU incorporation, consistent with the lack of any significant effect on tumor size. This observation is at variance with several reports that thiazolidinediones inhibit proliferation of human colon cancer cell lines in culture and in xenografts [9, 29–35]. The significance of this discrepancy requires additional investigation, but the data are consistent with the hypothesis that the suppressive effects of PPAR*γ* on established tumors may be due to inhibition of tumor progression, rather than inhibition of tumor growth.

On balance, the data seem unambiguously clear in one respect: PPAR*γ* suppresses colon carcinogenesis in mice. The primary effect appears to be inhibition of some early stage in transformation. The ability of PPAR*γ* to block early stage transformation is presumably due to some effect on normal colonic epithelial cells, which emphasizes the importance of understanding the functions of this receptor in the normal colonic epithelium. It is also clear that whereas PPAR*γ* inhibits proliferation of normal intestinal epithelial cells, this response is attenuated early in transformation. Although the antiproliferative effects of PPAR*γ* appear to be lost in cells that have either germ line or somatic mutations in *β*-catenin signaling, the data indicate that this receptor still retains the ability to inhibit tumor progression, at least in AOM-induced tumors. Finally, we submit that there is very little solid evidence that PPAR*γ* promotes colon carcinogenesis under any pharmacologically relevant conditions.

The role of PPAR*γ* as a suppressor of colon carcinogenesis in rodents is beyond question, but the evidence that PPAR*γ* is a colon cancer suppressor in humans is not so compelling. Although an early report indicated that loss-of-function mutations in PPAR*γ* were common in colon cancer [36], this claim has not subsequently been confirmed [37]. It has been reported that a polymorphism in codon 12 of PPAR*γ* is associated with colon cancer risk [38]. However, this polymorphism is manifest in PPAR*γ*2, which is expressed at relatively low levels in the colonic epithelium [3, 4], and not in the major colonic PPAR*γ* isoform, PPAR*γ*1 which differs in N-terminal sequence from PPAR*γ*2. PPAR*γ* expression is reduced in ulcerative colitis [39] and acromegaly [40], two conditions that predispose to colon cancer; and one might extrapolate from data with hemizygous knockout mice [41] to postulate that a reduction in PPAR*γ* expression increases the likelihood of transformation in the human colon. However, the evidence in support of such a conclusion is not very strong. Finally, a small phase II trial in which troglitazone was used to treat patients with late stage metastatic colon cancer produced no objective response [42]. One might argue that lack of response in this case was due to the rather low potency of the agonist or the very advanced stage of cancer in these patients. Alternatively, one might point to the data in mice, which indicate that PPAR*γ* has little or no effect on growth of established colon tumors in AOM-treated mice [20].

The best evidence for a tumor suppressive role of PPAR*γ* in humans comes from studies of established human colon cancer cell lines [9, 29–31, 33–35, 43]. Some of these cells exhibit growth arrest in culture when treated with thiazolidinediones, and growth of colon cancer xenografts has also been observed in thiazolidinedione-treated mice. However, many (in our experience most) human colon cancer cell lines are resistant to growth inhibition by concentrations of thiazolidinediones that are sufficient to maximally activate PPAR*γ*. Such observations raise two important questions: why are some colon cancer cell lines resistant to PPAR*γ* agonists? And to what extent are the effects of thiazolidinediones dependent upon PPAR*γ* expression and/or activity? Both of these questions are significant in terms of the use of thiazolidinediones as chemotherapeutic agents in colon cancer.

The most compelling case for pharmacological application of PPAR*γ* agonists in colon cancer is as a preventive agent. Clearly, PPAR*γ* is preventive in mouse models of sporadic colon cancer. However, the legal climate in USA at this time does not favor the development of chemopreventive drugs, particularly those that may have cardiovascular side effects [44]. These considerations do not preclude a prophylactic use for PPAR*γ* agonists. Patients with large numbers of ACF are at increased risk for development of subsequent colon cancers [45–47], and it is plausible that short-term treatment with PPAR*γ* agonists might result in a decrease in ACFs and a subsequent reduction in colon cancer risk. From a clinical standpoint, it is therefore important to ascertain if activation of PPAR*γ* in humans suppresses ACF formation, as has been observed in AOM-treated mice.

Finally, it is the case that several million individuals are taking thiazolidinediones for management of type II diabetes. It would seem that an epidemiological analysis of these individuals might, once and for all, determine if PPAR*γ* agonists have chemopreventive efficacy. Our initial attempts to collect data on time to formation of a second polyp in patients who are taking thiazolidinediones indicates that there are great many confounding variables, which limit the power of such an analysis and may account for the fact that few comprehensive anaylses of the effects of PPAR*γ* agonists on colon cancer incidence have been reported [48].

In summary, we conclude that both genetic and pharmacological data indicate that PPAR*γ* suppresses some early stage in transformation of colonic epithelial cells. We submit that the balance of evidence is inconsistent with the hypothesis that PPAR*γ* promotes colon carcinogenesis under any circumstances. Although it is unlikely that PPAR*γ* agonists will ever be developed as chemopreventive agents, it is plausible that we may, by studying the mechanism of action of PPAR*γ*, elucidate downstream effectors that may be chemopreventive targets. Furthermore, it is possible that PPAR*γ* agonists may have clinical applicability in prophylactic management of individuals who are at increased risk of colon cancer due to large numbers of ACFs.

## 4. PPAR*γ* AND INFLAMMATORY BOWEL DISEASE

PPAR*γ* has become a potential pharmacological target for treatment of inflammatory diseases of the colon, particularly ulcerative colitis (UC). PPAR*γ* is highly expressed in both colonic epithelial cells and macrophages critical for innate immunity and gut homeostasis. Over the past decade, in vitroand in vivo studies have defined an anti-inflammatory role for macrophage and epithelial PPAR*γ* in regulating colonic inflammation. Studies utilizing in vitro cell culture models have established that thiazolidinedione PPAR*γ* agonists can reduce NF*κ*B activation and inflammatory gene expression in colonic epithelial cells [49, 50], macrophages [51–54], dendritic cells [55–58], and T-cells [59, 60]. However, the magnitude by which thiazolidinediones reduce inflammatory gene expression and act directly through PPAR*γ* has been confusing. The anti-inflammatory effects of thiazolidinediones vary among studies due to differences in cell models, the concentration, duration, and type of thiazolidinedione (rosiglitazone, pioglitazone) used, as well as the context of inflammation studied (i.e., inducing agent-LPS, TNF-*α*, etc. [61]). More importantly, thiazolidinediones can reduce inflammation in both a PPAR*γ* dependent and independent manner, with the latter resulting from the use of high concentrations [62, 63]. Despite confusion, it has been generally accepted that thiazolidinediones can reduce inflammatory gene expression via PPAR*γ* in epithelial and immune cells when used at appropriate concentrations.

Perhaps the strongest evidence for an anti-inflammatory role of PPAR*γ* comes from landmark studies indicating that heterozygous PPAR*γ* deficient mice were more susceptible to DSS- and TNBS-induced colitis [64, 65]. DSS-induced colitis, in particular, is an acute inflammation model primarily driven by epithelial disruption and macrophage infiltration. This data indicates that PPAR*γ* expression in certain cell types of the colon plays an anti-inflammatory role. Recent studies elaborated on these findings by showing that mice deficient in PPAR*γ* expression in epithelial cells and macrophages displayed increased proinflammatory gene expression and susceptibility to DSS colitis [66, 67]. These findings suggest that PPAR*γ* expression in at least two cell types, epithelial and macrophage, can protect against at least one model of acute colitis (DSS). Questions remain regarding the importance of macrophage and epithelial PPAR*γ* in other models of acute and chronic colitis. For example, what is the role of macrophage or epithelial PPAR*γ* in a chronic colitis model driven by T-cells? Likewise, little is known about the role of dendritic and T-cell specific expression of PPAR*γ* in colitis, as PPAR*γ* knockout animals currently do not exist for these cell types. Experimental models of colitis can be initiated by distinct mechanisms and driven by infiltration of different cell types, both epithelial and immune [68–70]. Given that no single model accurately mimics human colitis, much remains to be understood regarding the tissue specific importance of PPAR*γ* in controlling gut inflammation. Furthermore, the importance of tissue specific PPAR*γ* expression may depend largely on the model of colitis examined, emphasizing the need to utilize multiple models to accurately represent manifestations of human colitis. Collectively, knockout animals have confirmed a potential protective role of PPAR*γ* in colitis but additional research is still required to understand fully how tissue specific PPAR*γ* expression influences different manifestations of colonic inflammation.

Based on the evidence that (a) thiazolidinediones can suppress inflammatory gene expression in vitro and (b) PPAR*γ* expression protects against the development of colitis in several animal models, it seems logical that PPAR*γ* might be a good target for treatment of gut inflammation. In reality, the preventative and therapeutic efficacy of targeting PPAR*γ* with thiazolidinediones for treatment of colitis is debatable. It remains unclear what phase, if any, would be best for targeting PPAR*γ* with thiazolidinediones for treatment of colitis: during the initiation, low grade, moderate, high grade, or remission phases of colitis. Our knowledge is largely based on animal studies in which acute preventive doses of thiazolidinediones were administered before the initiation of colitis. When given acutely (0–3 days) before inducing stimuli (i.e., DSS or TNBS) [50, 64–67, 71–73] or in the early life stages of animals that develop spontaneous cancer (IL-10^−/−^ mice) [74], thiazolidinediones provide beneficial effects in the amelioration of inflammation. These data indicate that at least one mode of PPAR*γ* action is to suppress the initiation of colitis, and suggest that thiazolidinediones may be a useful chemopreventative agent for the treatment of colitis. However, given their potential cardiovascular side effects, it is unlikely that thiazolidinediones would be approved as a chronic preventive agent for the management of gut inflammation. Moreover, the effectiveness of long term-preventative thiazolidinedione treatment on suppressing gut inflammation remains to be fully established. A recent report indicates that long-term treatment of mice with rosiglitazone exacerbated DSS-induced colitis [75], raising concerns about the preventative use of thiazolidinediones in gut inflammation.

The alternative scenario is that thiazolidinediones alone or in combination with anti-inflammatory/immunosuppressive drugs may be used to therapeutically target active inflammation. However, data from human and animal studies regarding the usefulness of therapeutic doses of thiazolidinediones in the treatment of colitis are inconsistent. When given after the initiation of DSS colitis, several studies found that thiazolidinediones had little or no value in improving colitis symptoms [50, 65, 73]. Likewise, therapeutic doses of thiazolidinediones given after established inflammation in the IL-10^−/−^ model of colitis provided no value [74]. Similar results were observed in a small open end clinical trial in which patients with moderate colitis receiving rosiglitazone experienced only modest improvement [76], however interpretation of these results are difficult as the trial lacked a proper control group. In contrast, a few studies have reported that therapeutic doses of rosiglitazone improved colitis symptoms in DSS and TNBS colitis [64, 77, 78]. Recently, a multicenter, randomized, double blind placebo-controlled trial for treatment of mild to moderately active UC with rosiglitazone showed clinical response in 44% of patients [79]. However, endoscopic remission rates were not significantly different. The discrepancies in thiazolidinedione effectiveness may reside with the doses of thiazolidinediones administered, with the magnitude of inflammation at the time of thiazolidinedione administration, or differences in scoring. Alternatively, the anti-inflammatory actions of thiazolidinediones may be inhibited or not strong enough to suppress inflammation during active colitis. Most likely, however, the lack of therapeutic efficacy of thiazolidinediones during active colitis can be explained by a loss of PPAR*γ* expression and/or activity that coincides with the level of inflammation. Indeed, PPAR*γ* levels have been shown to be downregulated in epithelial cells [39] and macrophages [73] during colitis. Moreover, Katayama et al. showed that the lack of therapeutic responsiveness to thiazolidinediones during colitis could be restored by adenoviral-mediated reexpression of PPAR*γ* in the colon [73]. In addition, Necela et al. showed that NF*κ*B drives down PPAR*γ* expression in response to lipopolysaccharide, thereby obviating the actions of PPAR*γ* and promoting an inflammatory state in macrophages [80].

In summary, although the current data supports a role for PPAR*γ* expression and activation in epithelial and immune cell types in the control of colonic inflammation, it remains unclear whether targeting PPAR*γ* with thiazolidinediones will be an effective strategy for treating gut inflammation. While animal models suggest a favorable role of thiazolidinediones in chemoprevention, the efficacy and safety of long-term use of thiazolidinediones as preventative agents or maintenance therapy in UC patients remains to be assessed. Likewise, the use of thiazolidinediones as therapeutic agents for treatment of colitis is controversial. It remains unclear during what clinical phase of inflammation thiazolidinediones would be most effective for treating UC patients or which patients would be most likely to benefit from treatment. Moreover, there is considerable concern regarding whether the adverse effects of thiazolidinediones would outweigh the potential benefit for patients with UC. In general, the majority of studies are in agreement that thiazolidinediones may be better exploited for therapeutic treatment of mild-moderate active colitis rather than during severe inflammation. Our understanding of the preventative and therapeutic potential of targeting PPAR*γ* with thiazolidinediones is largely limited by several factors, including the lack of (a) experimental usage of distinct models of colitis, (b) understanding of tissue specific roles of PPAR*γ* in different models of colonic inflammation, (c) understanding of factors affecting thiazolidinedione efficacy (PPAR*γ* levels, etc.), (d) understanding of the mechanism of the anti-inflammatory actions of PPAR*γ*, and (e) understanding of the adverse effects of short- and long-term use of thiazolidinediones during inflammation. Additional animal and clinical studies should resolve these discrepancies and provide further insight into the appropriate use, preventative or therapeutic, of thiazolidinediones for treatment of gut inflammation.
